# μ-α-Methyl­glutarato-bis­{aqua­[bis­(2-pyridylcarbon­yl)aminato]copper(II)} trihydrate

**DOI:** 10.1107/S1600536808034570

**Published:** 2008-10-31

**Authors:** Hong-Zhen Xie, Wei-Juan Pan, Wei Xu, Jian-Li Lin

**Affiliations:** a State Key Laboratory Base of Novel Functional Materials and Preparation Science, Faculty of Materials Science and Chemical Engineering, Ningbo University, Ningbo, Zhejiang 315211, People’s Republic of China

## Abstract

In the title compound, [Cu_2_(C_12_H_8_N_3_O_2_)_2_(C_6_H_8_O_4_)(H_2_O)_2_]·3H_2_O, both crystallographically independent Cu atoms are in similar distorted square-pyramidal coordination environments. The dinuclear complex mol­ecules are assembled into one-dimensional supra­molecular chains extending in the [100] direction by hydrogen bonds. Inter­chain hydrogen bonds further link these chains into layers perpendicular to [001].

## Related literature

For general background, see: Kajiwara *et al.* (2002 ▶); Kamiyama *et al.* (2000 ▶); Kooijman *et al.* (2006 ▶); Lescouezec *et al.* (2005 ▶); Ohkoshi & Hashimoto (1999 ▶); Ohkoshi *et al.* (1998 ▶); Smolin & Rapoport (1959 ▶); Toma *et al.* (2005 ▶);  Yamamoto *et al.* (1998 ▶); Zheng *et al.* (2006 ▶).  For related structures, see: Wei *et al.* (2002 ▶).
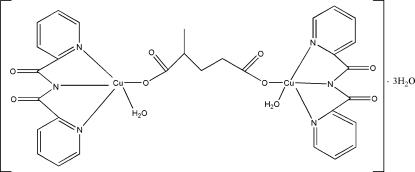


## Experimental

### 

#### Crystal data

[Cu_2_(C_12_H_8_N_3_O_2_)_2_(C_6_H_8_O_4_)(H_2_O)_2_]·3H_2_O
*M* *_r_* = 813.71
Orthorhombic, 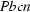

*a* = 7.2712 (15) Å
*b* = 26.910 (5) Å
*c* = 34.207 (7) Å
*V* = 6693 (2) Å^3^
*Z* = 8
Mo *K*α radiation
μ = 1.35 mm^−1^
*T* = 293 (2) K
0.31 × 0.28 × 0.18 mm

#### Data collection

Rigaku R-AXIS RAPID diffractometer
Absorption correction: multi-scan (*ABSCOR*; Higashi, 1995 ▶) *T*_min_ = 0.502, *T*_max_ = 0.547 (expected range = 0.720–0.785)
55287 measured reflections
7670 independent reflections
5714 reflections with *I* > 2σ(*I*)
*R*_int_ = 0.045

#### Refinement

*R*[*F* ^2^ > 2σ(*F* ^2^)] = 0.039
*wR*(*F* ^2^) = 0.108
*S* = 1.05
7670 reflections
461 parameters
H-atom parameters constrained
Δρ_max_ = 0.48 e Å^−3^
Δρ_min_ = −0.26 e Å^−3^

### 

Data collection: *RAPID-AUTO* (Rigaku, 1998 ▶); cell refinement: *RAPID-AUTO*; data reduction: *CrystalStructure* (Rigaku/MSC, 2002 ▶); program(s) used to solve structure: *SHELXS97* (Sheldrick, 2008 ▶); program(s) used to refine structure: *SHELXL97* (Sheldrick, 2008 ▶); molecular graphics: *ORTEPII* (Johnson, 1976 ▶); software used to prepare material for publication: *SHELXL97*.

## Supplementary Material

Crystal structure: contains datablocks global, I. DOI: 10.1107/S1600536808034570/cs2084sup1.cif

Structure factors: contains datablocks I. DOI: 10.1107/S1600536808034570/cs2084Isup2.hkl

Additional supplementary materials:  crystallographic information; 3D view; checkCIF report

## Figures and Tables

**Table 1:** Hydrogen-bond geometry (Å, °)

*D*—H⋯*A*	*D*—H	H⋯*A*	*D*⋯*A*	*D*—H⋯*A*
O3—H3*A*⋯O5^i^	0.89	2.06	2.835 (4)	144
O3—H3*B*⋯O12^i^	0.82	1.94	2.740 (4)	164
O6—H6*A*⋯O8^i^	0.80	2.04	2.819 (4)	165
O6—H6*B*⋯O1^ii^	0.82	2.00	2.822 (4)	179
O11—H11*A*⋯O13	0.962	1.94	2.808 (4)	150
O11—H11*B*⋯O2^iii^	0.910	2.17	2.968 (4)	146
O11—H11*B*⋯O1^iii^	0.910	2.41	3.129 (4)	136
O12—H12*A*⋯O10^iv^	0.88	2.09	2.907 (4)	154
O12—H12*B*⋯O9^v^	0.88	2.10	2.935 (4)	159
O12—H12*B*⋯O10^v^	0.88	2.53	3.108 (4)	124
O13—H13*B*⋯O7^vi^	0.820	2.13	2.933 (4)	168

Symmetry codes: (i) 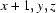; (ii) 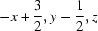; (iii) 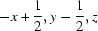; (iv) 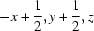; (v) 
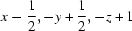
; (vi) 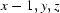.

**Fig. 1.**
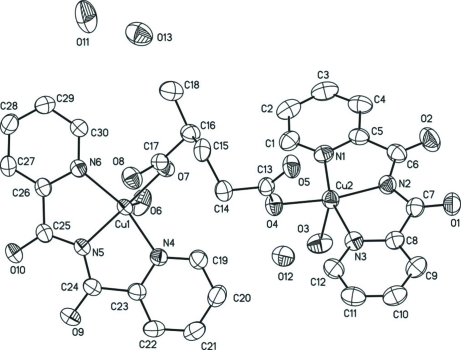
ORTEP view of the title complex with displacement ellipsoids drawn at the 45% probability level, H atoms omitted for visibility.

**Fig. 2.**
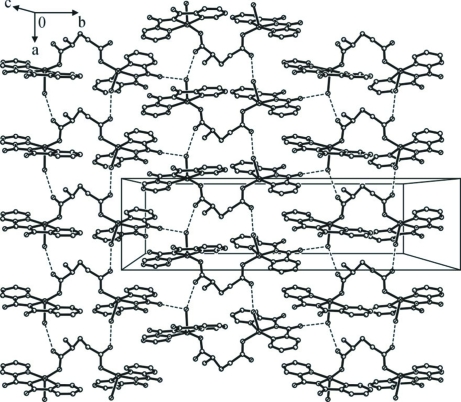
Two-dimensional supramolecular layer in the crystal perpendicular to the [001]direction with H-atoms omitted, broken lines indicate H-bridges.

## supplementary crystallographic information

### Comment

Chemistry of multi-metal-centered complexes attracted much attention because they often show interesting properties induced by direct and indirect *M*—*M* interactions such as magnetism (Ohkoshi & Hashimoto, 1999), conductivity (Yamamoto *et al.*, 1998) and photoactivity (Ohkoshi, *et al.*, 1998). Generally, the utilization of multidentate O– and N-donor ligands is an effective strategy to construct this kind of complex molecules. Hence, *bpca *(bis(2-pyridylcarbonyl)aminato) ligand is often used to prepare polynuclear complexes, (Lescouezec *et al.*, 2005; Kamiyama *et al.*, 2000; Kooijman *et al.*, 2006; Kajiwara *et al.*, 2002). However, investigations of the combination of *bpca* and dicarboxylate anions to design polynuclear ligands is limited. A novel dinuclear complex [Cu_2_(C_10_H_8_N_3_O_2_)_2_(C_6_H_8_O_4_)(H_2_O)_2_].3H_2_O was conceived as described.

*Tptz* (2,4,6-Tripyridyl-1,3,5-triazine) will only hydrolyse in the presence of concentrated mineral acids and temperatures above 150°C (Smolin & Rapoport, 1959) or in the presence of Cu^2+^ ions under mild conditions (Toma *et al.*, 2005). In absence of any acids reaction of *tptz* and Cu^2+^ produced [Cu(*bpca*)(*tca*)].2H_2_O (*tca*: 2-pyridinecarboxylate) (Zheng *et al.*, 2006). In the title compound *tptz* undergoes hydrolysis in the presence of α-methylglutaric acid.

The title crystal structure contains solvate water molecules and the dinuclear [Cu_2_(C_10_H_8_N_3_O_2_)_2_(C_6_H_8_O_4_) (H_2_O)_2_] complex (Fig 1). Both Cu atoms within the complex appear in similar square pyramidal coordination environments with the three N atoms of a bis(2-pyridylcarbonyl)amine (*bpca*) ligand and one O atoms of the α-methylglutarato ligand situtated at basal corners and one O atoms from the aqua ligand at apical position with normal Cu—N and Cu—O bond lengths (Wei *et al.*, 2002). The Cu—O bond 2.273 (2) Å and 2.272 (2) Å is slightly longer than that of basal ones which vary from 1.939 (2) Å to 2.015 (2) Å. Through intermolecular hydrogen bonds between the uncoordinated carboxylate O atoms and coordinated aqua ligand [O3—O5^i ^= 2.835 (2) Å and O6—O8^i ^= 2.819 (2) Å; symmetry code: (i) *x* + 1, *y*, *z*], respectively, the dinuclear complex molecules [Cu_2_(C_10_H_8_N_3_O_2_)_2_(C_6_H_8_O_4_)(H_2_O)_2_] are assembled into infinite chains extending in the [100] directions. The resulting chains are further interlinked into two-dimensional layers, perpendicular to the [001] direction, by interchain hydrogen bonds between the O1 of a *bpca* ligand and O6 of an aqua ligand (Fig. 2). The two-dimensional layers are stacked parallel and the solvate water molecules are sandwiched between them. Extensive hydrogen bonding exist between the included water molecules and the carbonyl O atoms of α-methylglutarato ligands or between the carboxylate O atoms of the α-methylglutarato ligand (Table 1).

### Experimental

Addition of 2.0 ml (1.0 *M*) NaOH to a stirred aqueous solution of CuCl_2_.2H_2_O (0.172 g, 1.0 mmol) yielded a blue precipitate. After centrifugation, the blue precipitate was subsequently added to a strirred solution of *tptz* (0.312 g, 1.0 mmol) and α-methylglutaric acid (0.146 g, 1.0 mmol) in 20 ml CH_3_OH–H_2_O (1:1 *v*/*v*). The resulting blue solution (pH = 6.13) was kept at room temperature and slow solvent evaporation afforded blue crystals (yield: 45% based on the initial CuCl_2_.2H_2_O input).

### Refinement

H atoms bonded to C atoms were placed in geometrically calulated positons and refined using a riding moldel, with *U*_iso_(H) = 1.2Ueq(C). Water H atoms were found in difference Fourier synthesis and refined with th O—H distances fixed as initially found, with *U*_iso_(H) = 1.2Ueq(O).

### Crystal data

**Table:**

[Cu_2_(C_12_H_8_N_3_O_2_)_2_(C_6_H_8_O_4_)(H_2_O)_2_]·3H_2_O	*F*(000) = 3344
*M**_r_* = 813.71	*D*_x_ = 1.615 Mg m^−^^3^
Orthorhombic, *P**b**c**n*	Mo *K*α radiation, λ = 0.71073 Å
Hall symbol: -P 2n 2ab	Cell parameters from 37378 reflections
*a* = 7.2712 (15) Å	θ = 3.0–27.5°
*b* = 26.910 (5) Å	µ = 1.35 mm^−^^1^
*c* = 34.207 (7) Å	*T* = 293 K
*V* = 6693 (2) Å^3^	Prism, blue
*Z* = 8	0.31 × 0.28 × 0.18 mm

### Data collection

**Table:**

Rigaku R-AXIS RAPID diffractometer	7670 independent reflections
Radiation source: fine-focus sealed tube	5714 reflections with *I* > 2σ(*I*)
graphite	*R*_int_ = 0.045
Detector resolution: 0 pixels mm^-1^	θ_max_ = 27.5°, θ_min_ = 3.0°
ω scans	*h* = −9→9
Absorption correction: multi-scan (ABSCOR; Higashi, 1995)	*k* = −34→34
*T*_min_ = 0.502, *T*_max_ = 0.547	*l* = −37→44
55287 measured reflections	

### Refinement

**Table:**

Refinement on *F*^2^	Primary atom site location: structure-invariant direct methods
Least-squares matrix: full	Secondary atom site location: difference Fourier map
*R*[*F*^2^ > 2σ(*F*^2^)] = 0.039	Hydrogen site location: inferred from neighbouring sites
*wR*(*F*^2^) = 0.108	H-atom parameters constrained
*S* = 1.05	*w* = 1/[σ^2^(*F*_o_^2^) + (0.0539*P*)^2^ + 2.9935*P*] where *P* = (*F*_o_^2^ + 2*F*_c_^2^)/3
7670 reflections	(Δ/σ)_max_ = 0.001
461 parameters	Δρ_max_ = 0.48 e Å^−^^3^
0 restraints	Δρ_min_ = −0.26 e Å^−^^3^

### Fractional atomic coordinates and isotropic or equivalent isotropic displacement parameters (Å^2^)

**Table:**

	*x*	*y*	*z*	*U*_iso_*/*U*_eq_	
Cu1	0.54934 (4)	0.175121 (11)	0.399089 (8)	0.03535 (9)	
Cu2	0.53997 (4)	0.428788 (12)	0.359585 (9)	0.03870 (10)	
N1	0.6355 (3)	0.39906 (8)	0.30992 (6)	0.0419 (5)	
N2	0.5980 (3)	0.49025 (8)	0.33255 (6)	0.0419 (5)	
N3	0.4645 (3)	0.47635 (8)	0.40161 (6)	0.0407 (5)	
N4	0.6124 (3)	0.22696 (8)	0.43940 (6)	0.0390 (5)	
N5	0.5884 (3)	0.13085 (8)	0.44302 (6)	0.0406 (5)	
N6	0.4822 (3)	0.11127 (8)	0.37233 (6)	0.0373 (5)	
O1	0.6058 (3)	0.57559 (7)	0.34103 (6)	0.0569 (5)	
O2	0.7173 (4)	0.51996 (9)	0.27399 (6)	0.0710 (7)	
O3	0.8095 (3)	0.40705 (9)	0.38851 (6)	0.0580 (5)	
H3A	0.9077	0.3911	0.3792	0.070*	
H3B	0.8148	0.4194	0.4104	0.070*	
O4	0.4229 (2)	0.36961 (7)	0.38088 (6)	0.0455 (4)	
O5	0.1669 (3)	0.40030 (7)	0.35585 (6)	0.0520 (5)	
O6	0.8389 (3)	0.17044 (8)	0.37424 (7)	0.0631 (6)	
H6A	0.9344	0.1804	0.3830	0.076*	
H6B	0.8544	0.1431	0.3642	0.076*	
O7	0.4551 (2)	0.22091 (7)	0.35979 (5)	0.0422 (4)	
O8	0.1987 (3)	0.20334 (8)	0.39062 (6)	0.0545 (5)	
O9	0.6702 (4)	0.13072 (8)	0.50887 (6)	0.0742 (7)	
O10	0.6010 (3)	0.04711 (7)	0.45907 (6)	0.0548 (5)	
O11	−0.1837 (5)	0.12871 (10)	0.26137 (10)	0.1035 (10)	
H11B	−0.1864	0.1000	0.2754	0.124*	
H11A	−0.2408	0.1608	0.2588	0.124*	
O12	−0.1091 (4)	0.43917 (9)	0.46254 (6)	0.0694 (6)	
H12A	−0.0701	0.4702	0.4636	0.083*	
H12B	−0.0190	0.4245	0.4753	0.083*	
O13	−0.3775 (4)	0.21512 (10)	0.28183 (7)	0.0788 (7)	
H13A	−0.4803	0.2103	0.2712	0.095*	
H13B	−0.4091	0.2186	0.3047	0.095*	
C1	0.6535 (4)	0.35111 (11)	0.30126 (9)	0.0494 (7)	
H1	0.6177	0.3276	0.3197	0.059*	
C2	0.7233 (4)	0.33506 (13)	0.26606 (9)	0.0587 (8)	
H2	0.7339	0.3013	0.2606	0.070*	
C3	0.7770 (5)	0.37017 (14)	0.23906 (9)	0.0634 (9)	
H3	0.8245	0.3603	0.2150	0.076*	
C4	0.7601 (4)	0.41966 (13)	0.24782 (8)	0.0560 (8)	
H4	0.7956	0.4437	0.2298	0.067*	
C5	0.6896 (4)	0.43331 (11)	0.28384 (7)	0.0427 (6)	
C6	0.6701 (4)	0.48686 (11)	0.29562 (8)	0.0461 (6)	
C7	0.5733 (3)	0.53352 (10)	0.35210 (8)	0.0424 (6)	
C8	0.4951 (3)	0.52436 (10)	0.39251 (8)	0.0405 (6)	
C9	0.4558 (4)	0.56223 (12)	0.41808 (9)	0.0536 (7)	
H9	0.4785	0.5951	0.4113	0.064*	
C10	0.3814 (5)	0.55019 (13)	0.45422 (9)	0.0592 (8)	
H10	0.3533	0.5751	0.4721	0.071*	
C11	0.3496 (4)	0.50144 (13)	0.46335 (8)	0.0569 (8)	
H11	0.2997	0.4928	0.4875	0.068*	
C12	0.3924 (4)	0.46541 (12)	0.43645 (8)	0.0498 (7)	
H12	0.3705	0.4323	0.4427	0.060*	
C13	0.2503 (4)	0.36850 (9)	0.37466 (7)	0.0383 (6)	
C14	0.1458 (4)	0.32513 (10)	0.39196 (8)	0.0464 (6)	
H14A	0.0544	0.3376	0.4101	0.056*	
H14B	0.2305	0.3043	0.4065	0.056*	
C15	0.0508 (4)	0.29403 (10)	0.36086 (9)	0.0495 (7)	
H15A	−0.0378	0.2722	0.3733	0.059*	
H15B	−0.0155	0.3158	0.3432	0.059*	
C16	0.1865 (4)	0.26321 (11)	0.33775 (8)	0.0454 (6)	
H16	0.2791	0.2855	0.3266	0.054*	
C17	0.2841 (4)	0.22620 (10)	0.36493 (7)	0.0401 (6)	
C18	0.0964 (5)	0.23415 (14)	0.30441 (10)	0.0677 (9)	
H18A	0.1890	0.2161	0.2903	0.081*	
H18B	0.0355	0.2568	0.2870	0.081*	
H18C	0.0083	0.2112	0.3150	0.081*	
C19	0.6176 (4)	0.27655 (10)	0.43521 (8)	0.0469 (6)	
H19	0.5975	0.2902	0.4106	0.056*	
C20	0.6518 (5)	0.30785 (11)	0.46620 (10)	0.0591 (8)	
H20	0.6551	0.3421	0.4625	0.071*	
C21	0.6809 (5)	0.28799 (12)	0.50268 (9)	0.0613 (8)	
H21	0.7008	0.3085	0.5241	0.074*	
C22	0.6800 (4)	0.23712 (12)	0.50692 (8)	0.0546 (7)	
H22	0.7025	0.2229	0.5312	0.065*	
C23	0.6456 (4)	0.20759 (10)	0.47502 (7)	0.0428 (6)	
C24	0.6377 (4)	0.15139 (10)	0.47802 (8)	0.0459 (6)	
C25	0.5724 (3)	0.08108 (10)	0.43633 (8)	0.0392 (6)	
C26	0.5099 (3)	0.07091 (9)	0.39491 (7)	0.0362 (5)	
C27	0.4783 (4)	0.02377 (10)	0.38124 (8)	0.0446 (6)	
H27	0.4976	−0.0036	0.3973	0.054*	
C28	0.4175 (4)	0.01755 (11)	0.34332 (9)	0.0503 (7)	
H28	0.3970	−0.0142	0.3334	0.060*	
C29	0.3879 (4)	0.05820 (11)	0.32057 (9)	0.0518 (7)	
H29	0.3450	0.0547	0.2951	0.062*	
C30	0.4223 (4)	0.10465 (10)	0.33579 (8)	0.0460 (6)	
H30	0.4032	0.1324	0.3201	0.055*	

### Atomic displacement parameters (Å^2^)

**Table:**

	*U*^11^	*U*^22^	*U*^33^	*U*^12^	*U*^13^	*U*^23^
Cu1	0.04081 (18)	0.03272 (16)	0.03252 (16)	0.00093 (12)	−0.00488 (13)	0.00500 (12)
Cu2	0.03813 (18)	0.03853 (18)	0.03945 (17)	−0.00216 (13)	0.00413 (13)	0.00180 (13)
N1	0.0352 (11)	0.0474 (13)	0.0429 (12)	−0.0011 (10)	−0.0028 (9)	−0.0040 (10)
N2	0.0423 (12)	0.0409 (12)	0.0424 (11)	−0.0042 (10)	0.0038 (10)	0.0018 (10)
N3	0.0343 (11)	0.0438 (12)	0.0441 (12)	0.0003 (9)	0.0023 (9)	0.0013 (10)
N4	0.0388 (11)	0.0392 (11)	0.0390 (11)	−0.0006 (9)	−0.0043 (9)	0.0026 (9)
N5	0.0498 (13)	0.0361 (11)	0.0358 (11)	−0.0021 (9)	−0.0064 (10)	0.0069 (9)
N6	0.0378 (11)	0.0363 (11)	0.0378 (11)	−0.0002 (9)	−0.0003 (9)	0.0045 (9)
O1	0.0696 (14)	0.0403 (11)	0.0607 (12)	−0.0068 (10)	0.0064 (11)	0.0039 (9)
O2	0.0964 (18)	0.0621 (14)	0.0544 (12)	−0.0039 (13)	0.0194 (13)	0.0150 (11)
O3	0.0429 (11)	0.0751 (14)	0.0562 (12)	0.0099 (10)	−0.0059 (9)	−0.0121 (11)
O4	0.0365 (10)	0.0407 (10)	0.0594 (12)	0.0002 (8)	0.0034 (9)	0.0073 (9)
O5	0.0456 (11)	0.0405 (10)	0.0699 (13)	0.0011 (9)	−0.0042 (10)	0.0123 (9)
O6	0.0454 (12)	0.0494 (12)	0.0944 (17)	−0.0048 (9)	0.0140 (12)	−0.0135 (11)
O7	0.0443 (10)	0.0427 (10)	0.0395 (9)	0.0084 (8)	−0.0055 (8)	0.0053 (8)
O8	0.0469 (11)	0.0563 (12)	0.0601 (12)	0.0039 (10)	−0.0005 (10)	0.0112 (10)
O9	0.120 (2)	0.0585 (13)	0.0436 (11)	−0.0118 (14)	−0.0271 (13)	0.0149 (10)
O10	0.0743 (14)	0.0414 (11)	0.0487 (11)	−0.0025 (10)	−0.0117 (10)	0.0150 (9)
O11	0.128 (3)	0.0629 (17)	0.120 (2)	0.0112 (17)	0.010 (2)	0.0282 (16)
O12	0.0928 (18)	0.0621 (14)	0.0531 (12)	0.0024 (13)	−0.0098 (12)	−0.0001 (11)
O13	0.0754 (16)	0.100 (2)	0.0607 (14)	−0.0013 (15)	0.0081 (12)	0.0096 (14)
C1	0.0420 (15)	0.0497 (16)	0.0565 (16)	−0.0013 (13)	−0.0056 (13)	−0.0061 (13)
C2	0.0512 (17)	0.063 (2)	0.0615 (19)	0.0038 (15)	−0.0064 (15)	−0.0207 (16)
C3	0.0538 (18)	0.086 (3)	0.0502 (17)	0.0043 (17)	−0.0012 (15)	−0.0222 (17)
C4	0.0498 (16)	0.080 (2)	0.0383 (14)	−0.0017 (16)	0.0020 (13)	−0.0009 (15)
C5	0.0353 (13)	0.0568 (16)	0.0360 (12)	−0.0018 (12)	−0.0035 (11)	0.0009 (12)
C6	0.0408 (14)	0.0555 (17)	0.0421 (14)	−0.0031 (12)	0.0004 (12)	0.0079 (13)
C7	0.0344 (13)	0.0439 (15)	0.0489 (15)	−0.0047 (11)	0.0003 (11)	0.0006 (12)
C8	0.0312 (12)	0.0464 (15)	0.0440 (14)	−0.0023 (11)	−0.0009 (10)	−0.0019 (12)
C9	0.0493 (17)	0.0514 (17)	0.0601 (18)	−0.0007 (13)	0.0030 (14)	−0.0114 (14)
C10	0.0567 (18)	0.068 (2)	0.0533 (17)	0.0021 (16)	0.0031 (15)	−0.0180 (16)
C11	0.0513 (17)	0.079 (2)	0.0404 (14)	0.0042 (16)	0.0073 (13)	−0.0023 (15)
C12	0.0474 (15)	0.0566 (17)	0.0455 (15)	0.0016 (13)	0.0048 (13)	0.0056 (13)
C13	0.0440 (14)	0.0319 (13)	0.0388 (13)	0.0017 (11)	0.0061 (11)	−0.0013 (10)
C14	0.0473 (15)	0.0421 (15)	0.0496 (15)	−0.0018 (12)	0.0071 (13)	0.0056 (12)
C15	0.0400 (15)	0.0373 (14)	0.071 (2)	−0.0006 (12)	−0.0042 (14)	0.0079 (13)
C16	0.0416 (14)	0.0508 (16)	0.0438 (14)	−0.0005 (12)	−0.0076 (12)	0.0048 (12)
C17	0.0474 (15)	0.0376 (13)	0.0354 (12)	0.0008 (12)	−0.0051 (12)	−0.0009 (11)
C18	0.075 (2)	0.076 (2)	0.0524 (18)	−0.0053 (19)	−0.0135 (17)	0.0037 (17)
C19	0.0488 (15)	0.0430 (15)	0.0490 (15)	0.0003 (12)	−0.0070 (13)	0.0048 (12)
C20	0.066 (2)	0.0412 (16)	0.070 (2)	−0.0052 (14)	−0.0114 (17)	−0.0046 (14)
C21	0.071 (2)	0.0567 (19)	0.0564 (18)	−0.0087 (16)	−0.0156 (16)	−0.0119 (15)
C22	0.0607 (19)	0.0628 (19)	0.0402 (14)	−0.0070 (15)	−0.0134 (14)	−0.0012 (13)
C23	0.0436 (15)	0.0456 (15)	0.0391 (13)	−0.0039 (12)	−0.0060 (11)	0.0033 (11)
C24	0.0533 (16)	0.0468 (15)	0.0377 (13)	−0.0045 (13)	−0.0078 (12)	0.0079 (12)
C25	0.0361 (13)	0.0393 (13)	0.0421 (13)	−0.0007 (10)	0.0002 (11)	0.0078 (11)
C26	0.0307 (12)	0.0380 (13)	0.0400 (13)	−0.0010 (10)	0.0047 (10)	0.0047 (11)
C27	0.0493 (15)	0.0344 (13)	0.0502 (15)	−0.0023 (12)	0.0051 (13)	0.0052 (12)
C28	0.0556 (17)	0.0411 (15)	0.0541 (16)	−0.0073 (13)	0.0039 (14)	−0.0068 (13)
C29	0.0605 (18)	0.0534 (17)	0.0417 (14)	−0.0065 (14)	−0.0039 (14)	−0.0052 (13)
C30	0.0586 (17)	0.0401 (14)	0.0392 (14)	−0.0010 (13)	−0.0059 (12)	0.0041 (12)

### Geometric parameters (Å, °)

**Table:**

Cu1—N5	1.939 (2)	C3—H3	0.9300
Cu1—O7	1.9479 (17)	C4—C5	1.384 (4)
Cu1—N6	2.007 (2)	C4—H4	0.9300
Cu1—N4	2.014 (2)	C5—C6	1.503 (4)
Cu1—O6	2.274 (2)	C7—C8	1.515 (4)
Cu2—N2	1.941 (2)	C8—C9	1.373 (4)
Cu2—O4	1.9470 (19)	C9—C10	1.388 (4)
Cu2—N3	2.001 (2)	C9—H9	0.9300
Cu2—N1	2.002 (2)	C10—C11	1.368 (5)
Cu2—O3	2.272 (2)	C10—H10	0.9300
N1—C1	1.330 (4)	C11—C12	1.373 (4)
N1—C5	1.342 (3)	C11—H11	0.9300
N2—C7	1.355 (3)	C12—H12	0.9300
N2—C6	1.371 (3)	C13—C14	1.513 (4)
N3—C12	1.335 (3)	C14—C15	1.520 (4)
N3—C8	1.347 (4)	C14—H14A	0.9700
N4—C19	1.342 (3)	C14—H14B	0.9700
N4—C23	1.347 (3)	C15—C16	1.512 (4)
N5—C25	1.364 (3)	C15—H15A	0.9700
N5—C24	1.366 (3)	C15—H15B	0.9700
N6—C30	1.336 (3)	C16—C18	1.530 (4)
N6—C26	1.348 (3)	C16—C17	1.536 (4)
O1—C7	1.217 (3)	C16—H16	0.9800
O2—C6	1.208 (3)	C18—H18A	0.9600
O3—H3A	0.8924	C18—H18B	0.9600
O3—H3B	0.8203	C18—H18C	0.9600
O4—C13	1.274 (3)	C19—C20	1.376 (4)
O5—C13	1.230 (3)	C19—H19	0.9300
O6—H6A	0.8019	C20—C21	1.374 (4)
O6—H6B	0.8200	C20—H20	0.9300
O7—C17	1.264 (3)	C21—C22	1.377 (4)
O8—C17	1.239 (3)	C21—H21	0.9300
O9—C24	1.216 (3)	C22—C23	1.373 (4)
O10—C25	1.218 (3)	C22—H22	0.9300
O11—H11B	0.9101	C23—C24	1.517 (4)
O11—H11A	0.9623	C25—C26	1.513 (4)
O12—H12A	0.8813	C26—C27	1.371 (4)
O12—H12B	0.8800	C27—C28	1.380 (4)
O13—H13A	0.8412	C27—H27	0.9300
O13—H13B	0.8222	C28—C29	1.360 (4)
C1—C2	1.376 (4)	C28—H28	0.9300
C1—H1	0.9300	C29—C30	1.377 (4)
C2—C3	1.378 (5)	C29—H29	0.9300
C2—H2	0.9300	C30—H30	0.9300
C3—C4	1.370 (5)		
			
N5—Cu1—O7	167.20 (9)	C11—C10—C9	119.5 (3)
N5—Cu1—N6	82.13 (9)	C11—C10—H10	120.2
O7—Cu1—N6	98.13 (8)	C9—C10—H10	120.2
N5—Cu1—N4	82.04 (9)	C10—C11—C12	119.1 (3)
O7—Cu1—N4	96.56 (8)	C10—C11—H11	120.5
N6—Cu1—N4	163.82 (8)	C12—C11—H11	120.5
N5—Cu1—O6	96.91 (9)	N3—C12—C11	122.1 (3)
O7—Cu1—O6	95.88 (8)	N3—C12—H12	118.9
N6—Cu1—O6	90.41 (8)	C11—C12—H12	118.9
N4—Cu1—O6	94.80 (8)	O5—C13—O4	123.8 (2)
N2—Cu2—O4	165.95 (9)	O5—C13—C14	119.6 (2)
N2—Cu2—N3	81.77 (9)	O4—C13—C14	116.6 (2)
O4—Cu2—N3	97.74 (9)	C13—C14—C15	112.3 (2)
N2—Cu2—N1	82.00 (9)	C13—C14—H14A	109.1
O4—Cu2—N1	98.17 (9)	C15—C14—H14A	109.1
N3—Cu2—N1	163.76 (9)	C13—C14—H14B	109.1
N2—Cu2—O3	103.84 (9)	C15—C14—H14B	109.1
O4—Cu2—O3	90.20 (8)	H14A—C14—H14B	107.9
N3—Cu2—O3	95.07 (8)	C16—C15—C14	111.8 (2)
N1—Cu2—O3	88.13 (8)	C16—C15—H15A	109.3
C1—N1—C5	119.3 (2)	C14—C15—H15A	109.3
C1—N1—Cu2	127.6 (2)	C16—C15—H15B	109.3
C5—N1—Cu2	113.05 (18)	C14—C15—H15B	109.3
C7—N2—C6	124.2 (2)	H15A—C15—H15B	107.9
C7—N2—Cu2	117.93 (17)	C15—C16—C18	113.0 (3)
C6—N2—Cu2	117.74 (18)	C15—C16—C17	109.9 (2)
C12—N3—C8	118.8 (2)	C18—C16—C17	108.5 (2)
C12—N3—Cu2	127.4 (2)	C15—C16—H16	108.4
C8—N3—Cu2	113.71 (17)	C18—C16—H16	108.4
C19—N4—C23	118.4 (2)	C17—C16—H16	108.4
C19—N4—Cu1	128.44 (18)	O8—C17—O7	122.4 (2)
C23—N4—Cu1	113.08 (17)	O8—C17—C16	121.3 (2)
C25—N5—C24	124.5 (2)	O7—C17—C16	116.3 (2)
C25—N5—Cu1	117.44 (17)	C16—C18—H18A	109.5
C24—N5—Cu1	117.98 (17)	C16—C18—H18B	109.5
C30—N6—C26	118.5 (2)	H18A—C18—H18B	109.5
C30—N6—Cu1	128.33 (18)	C16—C18—H18C	109.5
C26—N6—Cu1	113.10 (17)	H18A—C18—H18C	109.5
Cu2—O3—H3A	131.2	H18B—C18—H18C	109.5
Cu2—O3—H3B	109.5	N4—C19—C20	122.1 (3)
H3A—O3—H3B	118.9	N4—C19—H19	119.0
C13—O4—Cu2	112.80 (16)	C20—C19—H19	119.0
Cu1—O6—H6A	130.1	C21—C20—C19	119.3 (3)
Cu1—O6—H6B	109.5	C21—C20—H20	120.4
H6A—O6—H6B	109.6	C19—C20—H20	120.4
C17—O7—Cu1	108.74 (16)	C20—C21—C22	118.8 (3)
H11B—O11—H11A	143.2	C20—C21—H21	120.6
H12A—O12—H12B	99.4	C22—C21—H21	120.6
H13A—O13—H13B	100.4	C23—C22—C21	119.5 (3)
N1—C1—C2	122.4 (3)	C23—C22—H22	120.2
N1—C1—H1	118.8	C21—C22—H22	120.2
C2—C1—H1	118.8	N4—C23—C22	121.9 (3)
C1—C2—C3	118.4 (3)	N4—C23—C24	116.1 (2)
C1—C2—H2	120.8	C22—C23—C24	122.0 (2)
C3—C2—H2	120.8	O9—C24—N5	128.8 (3)
C4—C3—C2	119.6 (3)	O9—C24—C23	120.5 (3)
C4—C3—H3	120.2	N5—C24—C23	110.7 (2)
C2—C3—H3	120.2	O10—C25—N5	128.0 (3)
C3—C4—C5	119.0 (3)	O10—C25—C26	120.9 (2)
C3—C4—H4	120.5	N5—C25—C26	111.1 (2)
C5—C4—H4	120.5	N6—C26—C27	121.7 (2)
N1—C5—C4	121.2 (3)	N6—C26—C25	115.8 (2)
N1—C5—C6	116.9 (2)	C27—C26—C25	122.5 (2)
C4—C5—C6	121.9 (3)	C26—C27—C28	119.1 (3)
O2—C6—N2	128.6 (3)	C26—C27—H27	120.5
O2—C6—C5	121.1 (3)	C28—C27—H27	120.5
N2—C6—C5	110.3 (2)	C29—C28—C27	119.4 (3)
O1—C7—N2	128.3 (3)	C29—C28—H28	120.3
O1—C7—C8	120.5 (3)	C27—C28—H28	120.3
N2—C7—C8	111.1 (2)	C28—C29—C30	119.0 (3)
N3—C8—C9	122.0 (3)	C28—C29—H29	120.5
N3—C8—C7	115.4 (2)	C30—C29—H29	120.5
C9—C8—C7	122.6 (3)	N6—C30—C29	122.3 (3)
C8—C9—C10	118.4 (3)	N6—C30—H30	118.9
C8—C9—H9	120.8	C29—C30—H30	118.9
C10—C9—H9	120.8		

### Hydrogen-bond geometry (Å, °)

**Table:**

*D*—H···*A*	*D*—H	H···*A*	*D*···*A*	*D*—H···*A*
O3—H3A···O5^i^	0.89	2.06	2.835 (4)	144
O3—H3B···O12^i^	0.82	1.94	2.740 (4)	164
O6—H6A···O8^i^	0.80	2.04	2.819 (4)	165
O6—H6B···O1^ii^	0.82	2.00	2.822 (4)	179
O11—H11A···O13	0.962	1.94	2.808 (4)	150
O11—H11B···O2^iii^	0.910	2.17	2.968 (4)	146
O11—H11B···O1^iii^	0.910	2.41	3.129 (4)	136
O12—H12A···O10^iv^	0.88	2.09	2.907 (4)	154
O12—H12B···O9^v^	0.88	2.10	2.935 (4)	159
O12—H12B···O10^v^	0.88	2.53	3.108 (4)	124
O13—H13B···O7^vi^	0.820	2.13	2.933 (4)	168

Symmetry codes: (i) *x*+1, *y*, *z*; (ii) −*x*+3/2, *y*−1/2, *z*; (iii) −*x*+1/2, *y*−1/2, *z*; (iv) −*x*+1/2, *y*+1/2, *z*; (v) *x*−1/2, −*y*+1/2, −*z*+1; (vi) *x*−1, *y*, *z*.
